# The post discharge stroke care services in Malaysia: a pilot analysis of self-reported practices of family medicine specialists at public health centres

**DOI:** 10.1186/1471-2458-12-S2-A1

**Published:** 2012-11-27

**Authors:** Aznida Firzah Abdul Aziz, Nor Azah Aziz, Saperi Sulong, Syed Mohamed Aljunid

**Affiliations:** 1 United Nations University-International Institute for Global Health, Universiti Kebangsaan Malaysia Medical Centre, Jalan Yaacob Latiff, 56000 Kuala Lumpur, Malaysia; 2 Department of Family Medicine, Universiti Kebangsaan Malaysia Medical Centre, Jalan Yaacob Latiff, 56000 Kuala Lumpur, Malaysia; 3 Health Information Department, Universiti Kebangsaan Malaysia Medical Centre, Jalan Yaacob Latiff, 56000 Kuala Lumpur, Malaysia

## Background

The care of post discharge stroke patients worldwide is fragmented. For developing countries, lack of guidelines upon discharge from tertiary care and access to specialised care complicates provision of optimal care. Albeit shortcomings, primary care continues to provide care in less than favourable circumstances. This study aimed to evaluate practices of Family Medicine Specialists (FMS) in managing post discharge stroke patients at primary care level.

## Materials and methods

A semi structured self-administered questionnaire was distributed among FMS servicing public funded health centres. The questionnaire assessed estimated stroke care burden, current service provision and opinion on service improvement. Descriptive and qualitative analyses were done.

## Results

Total of 59 completed questionnaires were analysed. For every 100 patients seen by FMS’ at public health centres each month, 2 patients have stroke. Median number of stroke patients seen per month was 5 (IQR 2-10). Fifty seven percent of the respondents estimated total stroke patients treated per year at each centre was less than 40 patients. As high as 72.4% did not have a standard care plan although 96.6% agreed one was needed and would improve quality of post stroke care. Patients seen were: discharged from tertiary care (88.1%), shared care plan with specialists (67.8%) and patients who developed stroke during follow up at primary care (64.4%). Follow-up was done at 8-12 weekly intervals (60.3%) with only 3.4% on as needed basis. Referrals ranked in order of frequency were to physiotherapy services, dietitian and speech and language pathologists in public facilities. The FMS perceived 4 important factors in managing stroke patients at primary care level; need for access to rehabilitation services, coordinated care between tertiary centres and primary care using multidisciplinary care approach, need for a stroke care guideline and availability of family and caregiver support services.

## Conclusions

Post discharge stroke care guidelines and access to rehabilitation services at primary care are needed for optimal delivery of care to patients residing at home in the community.

